# SMS-based interventions for improving child and adolescent vaccine coverage and timeliness: a systematic review

**DOI:** 10.1186/s12889-024-18900-4

**Published:** 2024-07-02

**Authors:** GE Currie, C McLeod, C Waddington, TL Snelling

**Affiliations:** 1 School of Public Health, Faculty of Medicine and Health, University of Sydney, Camperdown, NSW Australia; 2 Wesfarmers Centre of Vaccines and Infectious Diseases, Telethon Kids Institute, University of Western Australia, Crawley, WA Australia; 3 Department of Medicine, School of Clinical Medicine, University of Cambridge, Cambridge, UK; 4 School of Public Health, Curtin University, Bentley, WA Australia; 5 Menzies School of Health Research and Charles Darwin University, Casuarina, NT Australia

**Keywords:** Childhood vaccination, Immunisation, Coverage, Timeliness, SMS reminders, Text messages

## Abstract

**Background:**

The aim of this review was to investigate the impact of short message service (SMS)-based interventions on childhood and adolescent vaccine coverage and timeliness.

**Methods:**

A pre-defined search strategy was used to identify all relevant publications up until July 2022 from electronic databases. Reports of randomised trials written in English and involving children and adolescents less than 18 years old were included. The review was conducted in accordance with PRISMA guidelines.

**Results:**

Thirty randomised trials were identified. Most trials were conducted in high-income countries. There was marked heterogeneity between studies. SMS-based interventions were associated with small to moderate improvements in vaccine coverage and timeliness compared to no SMS reminder. Reminders with embedded education or which were combined with monetary incentives performed better than simple reminders in some settings.

**Conclusion:**

Some SMS-based interventions appear effective for improving child vaccine coverage and timeliness in some settings. Future studies should focus on identifying which features of SMS-based strategies, including the message content and timing, are determinants of effectiveness.

**Supplementary Information:**

The online version contains supplementary material available at 10.1186/s12889-024-18900-4.

## Background

Vaccinating children prevents an estimated 2.5 million deaths each year [1] and ensuring that vaccine coverage remains high is an important public health priority [2]. Despite this, global vaccine coverage was static over the last decade, and fell from 86% in 2019 to 83% in 2020 in the context of the COVID-19 pandemic, leaving an estimated 23 million infants under-vaccinated [3]. The reasons for under-vaccination are complex and multifactorial. Lack of the five ‘A’s—access, affordability, awareness, acceptance and activation—have been proposed as a taxonomy for the core contributing factors across a range of socio-geographical-cultural contexts [4]. Across the world, immunisation is largely coordinated at a population level, and typically as either national or state/provincial level programs [5]. Immunisation programs typically implement a fixed schedule of vaccination at specific age-based timepoints, although vaccines may also be scheduled to align with other events such as school or college entry and pregnancy.

Text messaging by short message service (SMS) via mobile (cellular) phones, has been used to deliver reminders to promote health behaviours, including for vaccination. The SMS content may assist to target specific barriers to vaccination like poor awareness, acceptance or access [6]. Compared to other communication channels, SMS is cheap, instantaneous, and less confrontational [7], and allows the recipient to attend to the message when convenient. Mobile phone coverage is now extensive in both developed and developing settings [8] across income levels [9], enabling broad capture of the population [10]. Although mobile network connectivity has rapidly expanded globally, uptake of health interventions driven through mobile phone technology (mHealth) have been slower in low-middle income settings compared to high income settings, likely due to limited availability of technical support and infrastructure investment to support scaling [11].

Three recent systematic reviews summarised research assessing the effect of SMS-based interventions on childhood vaccine coverage in low-income [12], low-middle income [13] and both high and low-income settings [14]. We sought to update these reviews with newly published research, including studies of adolescents due for vaccination, and including data relating to the effectiveness of SMS-based interventions on vaccine timeliness. A growing number of vaccines are now targeted toward adolescents, and they are a distinct demographic from children and adults. Adolescents are likely to fall somewhere between children and adults with respect to both the achieved uptake of recommended vaccines, and the extent to which they, versus their parents, are responsible for their healthcare decision-making. Furthermore, this group may interact with technology, and hence SMS reminders, differently from other groups.

### PICO statement

The aim of this systematic review was to examine: for parents of children or adolescents (< 18 years) eligible for a routine vaccination (P), what is the impact of SMS reminders (I) on vaccine coverage and timeliness (O), compared to standard care or other reminder methods (C).

## Methods

### Search strategy

This PROSPERO registered systematic review (CRD42016048290) was conducted in accordance with PRISMA guidelines [15]. We searched PubMed, Medline, Embase, Cochrane, Cumulative Index to Nursing and Allied Health Literature (CINHAL), PsycINFO and Web of Science for studies published through to July 2022 using the following search terms in a Boolean strategy: vaccination, immunization, immunisation, immunis*, immuniz*, immunis*, SMS, smartphone, telemedicine, mHealth, mobile health, short message service, cell phone, text messaging, text reminder and mobile phones (see *Appendix 1*). The search was limited to full-text studies written in English involving adolescents or parents of children less than 18 years old. Additional papers were identified through reference searching of peer reviewed manuscripts and grey literature.

### Eligibility criteria

We included randomised studies examining (i) the impact of SMS-based interventions on coverage and/or timeliness of child vaccines. We included studies that compared alternative SMS-based reminder strategies without a non-SMS control group (e.g. postcard reminders). We excluded studies where adjunctive interventions were also used (e.g. flyers or education) that i) did not report the effects of SMS-based reminders only or ii) where the control group did not receive the same adjunctive intervention as the SMS-based reminder group. We excluded randomised studies that did not randomise to a control arm. We excluded non-randomised studies (i.e. original observational studies) due to the availability of higher quality randomised studies, especially considering most SMS evaluations compare before-versus-after designs, and non-randomised studies introduce a high risk of confounding by temporal factors.

### Study definitions

Vaccine coverage was defined as the proportion of vaccine-eligible children within a study group who received all specified vaccine(s) within a defined time-period. Vaccine timeliness was defined as a measure of vaccine administration relative to the due date, either (i) the proportion vaccinated within a set period after the scheduled date or (ii) the time to vaccination after the scheduled date. Low-middle and high-income countries were categorised according to World Bank definitions [16] and analysed separately. The impact of SMS-based interventions on special interest groups or vaccines and whether interventions were issued as pre-call (prior to the due date), or recall (after the due date) were also analysed separately for vaccine coverage.

### Study selection, data analysis, and *bias*

Two reviewers (GC and CM) independently performed and screened the search output and reviewed potentially eligible full-text studies after removing duplicates. Studies were summarised by design, study population, intervention and comparator groups, outcomes and limitations. The primary reviewer (GC) performed study quality assessment using the National Heart Lung and Blood Institute (NHLBI) checklist for randomised trials [17]. Ten percent of data extraction and bias assessments were randomly selected and cross-checked for accuracy by the second reviewer (CM). Discrepancies between the primary reviewers were resolved by consensus, or where necessary by a third reviewer (TS). A meta-analysis was not performed owing to the marked heterogeneity of included studies. Findings are therefore described by narrative review.

## Results

### Search results

**Fig. 1:**
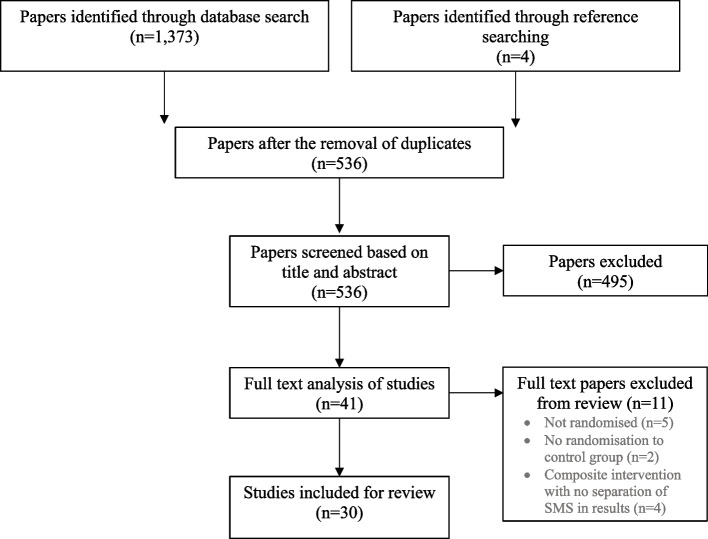
PRISMA flow diagram of the search results

A total of 536 publications were identified after removing duplicates; after screening abstracts, 44 papers were selected for full text review *(See *Fig. 1* for flow diagram)*. Of these, 30 met the inclusion criteria and were included in the final review (Table 1). Two trials were excluded from review as they did not assign participants to a control group, instead comparing recipients of different SMS reminders to no control [18] and non-enrolled parents in the study [19].

**Table 1:** Randomised Trials examining the effectiveness of SMS interventions

**Author/year**	Population	n	Intervention group	Comparator group	Outcome measure	Results	Limitations	Quality rating (Good, fair, poor)/assessment of bias
***Low-Middle Income***								
Bangure et al. (2015) [20]	Zimbabwe: mothers recruited following delivery of newborn	304	SMS reminders at 6, 10 and 14 wks	Routine education	Vaccination coverage and timeliness at 6, 10 and 14 wks	Coverage and timeliness increased at all time points in the intervention group (*p* < 0.001)	Unsure if blinding occurred to control performance bias	Fair
Chandir et al. (2022) [21]	Pakistan: parents of children < 2 years presenting for first vaccines	11,197	5 arms of differing mobile conditional cash transfers (mCCT) + SMS, SMS only	No SMS reminder	Full vaccination coverage at 12 months	High flat rate incentives + SMS (OR: 1.30, CI 1.11–1.51), High sharp rate incentives + SMS (OR: 1.27, CI 1.09–1.48) & SMS only (OR: 1.16, CI 1.00–1.34) superior compared to control	Some participants could not receive mCCTs due to mobile phone incompatibility	Good
Domek et al. (2019) [22]	Guatemala: parents of children between 6wks to 6mo who presented for their first vaccines	720	One SMS reminder sent 3 days, 2 days and 1 day prior to 2nd and 3rd vaccine visits	Routine care	Vaccination timeliness at 2, 4 and 6 months	Coverage similar across groups. Intervention group received vaccines on scheduled dates of visit 2 (42.2% vs 30.7%, p-0.001) and 3 (34% vs 27%, *p* = 0.05) and within 7 days of visit 2 (71% vs 63.5%, *p* = 0.03) compared to control	Vaccine shortages present in Guatemala during study so used attendance as proxy for status. Some errors in SMS system where not all participants were sent SMS	Fair
Domek et al. (2016) [23]	Guatemala: caregivers of children presenting for 1st vaccines at 8–14 wks	321	SMS reminders 1 wk prior to 2nd/3rd vaccines	Routine care	Vaccination coverage: completion of the primary immunisation series	Both intervention/control groups had high rates of vaccine completion (visit 2: 95 vs. 90% and visit 3: 84 vs. 81% respectively	Pilot study. Service interruptions	Fair
Eze et al. (2015) [24]	Nigeria: caregivers attending health clinics	905	SMS reminders to everyone in intervention group and additional recall SMS to parents who did not attend appointment	Routine care	Vaccination coverage and vaccination timeliness of receipt of DPT3 prior to 18th week	Intervention group DPT3 8.7% higher coverage and received DPT3 1.5 × earlier than controls (OR 1.47, CI 1.1–2.0, *p* = 0.009)	Inconsistent DPT product supply. Randomisation procedures did not account for mobile phone ownership	Poor
Gibson et al. (2017) [6]	Kenya: parents of newborns residing in rural villages	2,018	1) SMS only (3 and 1 day prior to scheduled vaccines at 6, 10 and 14 weeks & 9 months); 2) SMS + low monetary incentive; 3) SMS + higher monetary incentive	Routine care	Vaccination coverage: Proportion of fully immunised children at 12 months (including BCG). and Hepatitis B), measles and BCG vaccines. Vaccine coverage & vaccination timeliness (within 2 weeks) for pentavalent, polio and measles vaccines	SMS + higher monetary incentive group more likely to achieve primary outcome (RR 1.09, (1.02–1.16), *p* = 0.014). No difference between SMS only or SMS + low monetary incentive group. 2^0^ outcomes: Improved timeliness of measles vaccine seen in all 3 intervention groups but highest in SMS + high incentive group (RR 1.42 (1.23–1/65), *p* < 0.0001)	Incomplete information about whether reminders were received/read. Randomisation assignment cluster randomised by village at public ceremony. High baseline immunisation rates	Fair
Haji et al. (2016) [25]	Kenya: parents of children residing in low pentavalent coverage districts	1,116	1) SMS reminder; 2) sticker reminders	Routine care	Vaccination coverage: dropout rate (missing 2nd/3rd pentavalent vaccine doses) 2 weeks after scheduled visit for 3rd vaccine	SMS group 20% less likely to drop out compared to control (OR 0.2, CI 0.04–0.8)	Randomised at clinic level. Randomisation methods not detailed. No specification of intention to treat analysis	Poor
Ibraheem et al. (2021) [26]	Nigeria: mother-infant pairs present for first vaccination	560	1) SMS reminders; 2) Educational SMS; 3) Phone call reminders	Routine care (no reminder)	Vaccination coverage and vaccination timeliness	All intervention groups had higher completion rates compared to control. Timeliness of plain SMS (AOR 2.56, 1.96–3.35) and educational SMS (AOR: 2.44, 1.87–3.18) similar odds and superior compared to control. Calls superior to SMS	Reduced generalisability. Randomisation methods not described in detail. Demographics table not broken into randomised groups. No specification of intention to treat analysis	Fair
Kawakatsu et al. (2020) [27]	Nigeria: parents of children attending primary health centres	9,368	SMS reminder	Usual care (verbal and written reminders)	Vaccination coverage: Antenatal and family planning appointment attendance	SMS group had higher vaccine uptake compared to control (4.8–6% higher at all time points, *p* < 0.001) and more likely to receive vaccines (AOR: 1.17, 95% CI: 1.05–1.31)	Randomisation did not account for the appointment type so required further statistical adjustments	Good
Seth et al. (2018) [28]	India: pregnant women and parents of children < 24mo in rural India	608	1) SMS reminders + phone credit incentives; 2) SMS reminders only	Written reminder	Vaccination coverage for all required vaccines at study end Vaccination timeliness within 14 days of each vaccine	SMS + phone credit incentives group had higher vaccination coverage (RR 1.09, CI1.002–1.18, *p* = 0.04) and more timely vaccine receipt (40%) compared to SMS only and control	Low literacy level of study population. Study field staff not blinded to random allocation. No intention to treat analysis. No power calculations provided	Poor
Shinde et al. (2018) [29]	India: mothers of children 0–3 weeks old attending the maternity ward	125	SMS reminders	Immunisation card	Vaccination coverage at 10 wks	SMS reminder group had higher coverage at 10w compared to control (95% vs 77%, *p* = 0.011)	Low sample size. Randomisation concealment not described	Fair
***High-Income***								
Ahlers-Schmidt et al. (TRICKS) (2012) [30]	USA: parents of newborns discharged from hospital in Kansas	90	SMS reminder + appointment card	Appointment card	Vaccination coverage and timeliness of vaccines at 2, 4, 6 months	Greater numbers in intervention group received vaccines and on time, but not statistically significant	Pilot; small sample size. Selection bias (differed in income status) and attrition bias due to loss of phone service. Problematic software	Poor
Coleman et al. [31]	USA: Parents of preschool children in New York	57	SMS reminder	Written reminder	Influenza vaccine timeliness	Time to vaccination shorter in SMS group (42 days vs. 62 days; *p* < 0.05)	Small sample size. Performance and selection bias (randomised per patient preference & 8% had sibling in control group)	Poor
Gurfinkel et al. (2021) [32]	USA: parents of children due for initial or subsequent HPV doses in New York	37,003	1) SMS; 2) Autodial reminder	Usual care (no reminder)	HPV vaccination coverage for 1, 2 or 3rd dose. Timeliness to completion	No significant difference between groups for uptake or timeliness	Randomisation procedures not described in detail. Unknown if randomisation concealment occurred. Baseline characteristics of participants per arm not provided	Fair
Hofstetter et al. (2015) [33]	USA: parents of children due for 12-month check-up (9.5mo-10mo) in New York	2054	1) SMS appointment reminder (2 days prior to scheduled 1 year appointment); 2) × 3 scheduling reminders to book vaccination appointment AND SMS appointment reminder as above	Usual care (routine telephone reminder)	Vaccination coverage: 12-month appointment attendance Vaccination timeliness of MMR vaccine	No difference in MMR vaccination by 13 months between groups. Only in post-hoc for parents that had not booked an appointment prior to study (attendance rates: 62.1% vs. 54.7%, relative risk ratio 1.14 95% CI 1.04–1.24). More likely to have timely MMR vaccine (61.1% vs. 55.1%, relative risk ratio 1.11 95% CI 1.01–1.21)	Specific low-income, minority, Spanish-speaking families, limiting generalisability to other settings. No mention of intention to treat analysis or randomisation concealment	Fair
Hofstetter et al. (2015) [34]	USA: Low-income, minority parents of under-vaccinated children (6mo-17y) in New York	5462	1) Educational + interactive SMS; 2) educational only SMS	Telephone reminder	Influenza vaccination coverage Influenza vaccination timeliness	More children in educational & interactive group vaccinated than other two groups (38.5% vs. 35.3% vs. 34.8%; RRR: 1.09, 95% CI 1.002–1.19) & more timely vaccination than educational only (AHR = 0.90, 95% CI = 0.81–1.00) and standard care (adjusted hazards ratio = 0.88, 95% CI = 0.79–0.98)	Urban low-income participants, limiting generalisability to other settings. No mention of randomisation concealment	Good
Menzies et al. (2020) [35]	Australia: Parents of children due for routine childhood vaccines	1,594	(1) SMS reminder only; (2) Calendar only; (3) SMS reminder + calendar	No reminder	Vaccination coverage: 30 days within due date of 2,4,6,12 and 18 month vaccines	SMS reminders alone (RR 1.09, 95% CI 1.01–1.18) or in combination with a personalised calendar (1.11, CI 1.03–1.20) higher compared to control at 12 m endpoint only	Low sample size in 4 m timepoint. High compliance in control group compared to national statistics. No mention of randomisation concealment or blinded assessment. Major policy change required unplanned subgroup analysis	Good
Niederhauser et al. (2015) [36]	USA: mothers/neonates in Hawaii	57	SMS reminders sent 4 and 2 weeks prior to the 2, 4 and 6 mo. Vaccines	Sham SMS of age-appropriate newborn health messages sent at equivalent time point	Vaccination coverage: compliance with vaccinations + 7 and + 14 days post due-dates. Barriers SHOTS survey	At all assessment points (bar 1 time point), the control group had higher rates of vaccine compliance, although higher barriers in intervention group	Pilot; small sample size. Selection and attrition bias due to high drop out rate. Randomisation procedures not described in detail. Groups not equal at baseline. No intention to treat analysis performed	Poor
O’Grady et al. (2022) [37]	Australia: mothers of children attending primary care clinics	196	1) SMS reminder; 2) Educational SMS reminder	No reminder	Vaccination coverage at 7 months for 2, 4 and 6 month vaccines Vaccination timeliness in days	Improved vaccine coverage at all timepoints in educational SMS reminder compared to control (7mo ARR: 2.28 95% CI 1.05—4.94). There was no difference between simple SMS reminders and control. Timeliness: no differences between either SMS group and control	Low sample size. Groups not similar at baseline; authors state this did not impact results in their regression analysis	Good
O’Leary et al. (2015) [38]	USA: parents of adolescents attending 5 private, 2 public clinics in Colorado	4587	SMS bidirectional message (1) Clinic will call to schedule (2) Parent will call (3) STOP	No reminder	Vaccination coverage: receipt of all vaccinations and any vaccine	Intervention group more likely to receive all vaccinations (RR 1.29, 95% CI 1.12–1.5) and any vaccine (RR 1.36; 95% CI 1.2–1.54)	Didn’t directly compare unidirectional and bidirectional messaging	Good
Rand et al. (2015) [39]	USA: Adolescents 11–16 years with no prior HPV at 39 practices in New York	3812	SMS reminders (up to 4) to parents of adolescents for HPV	Sham SMS of general health messages	Vaccination coverage: receipt of HPV1, receipt of HPV2 & 3	No differences between groups for any dose. Post-hoc analysis for those able to receive message showed 30% HPV1 (HR 1.3, 95% CI 1–1.6)	Only half participants had a phone with SMS capability despite being randomised. Single centre. Planned stratified analysis limited by insufficient sample size. Unclear if lack of randomisation concealment impacted performance bias	Poor
Rand et al. (2017) [40]	USA: Parents of adolescents 11–16 years in 3 urban primary care clinics presenting for HPV 1 or 2 in New York	749	1) SMS reminder; 2) telephone calls	No reminder	Vaccination coverage as receipt of all 3 doses. Vaccination timelines: time to receipt of HPV vaccination	49% SMS vs. 40% controls received 3 HPV doses (*p* = 0.001). Time to receipt HPV3 greater in SMS group (HR 2.34, *p* < 0.001) and phone group (HR = 1.91, *p* = 0.007) who enrolled at time HPV1 vs. controls	Limited sample size. Didn’t directly compare SMS vs. phone reminders. Randomisation was based on parental preference of receiving SMS or phone	Fair
Szilagyi et al. (2020) [41]	USA: Parents of children in New York primary care practices	61,931	1) SMS reminder; 2) autodial reminder; 3) mailed reminders	No reminder	Influenza vaccine coverage within 6 months	No difference between SMS reminders and control group (27.6% vs 26.6%)	No mention of randomisation concealment or blinding procedures. No mention of how sample size was calculated	Good
Szilagyi et al. (2020) [41]	USA: Parents of children attending elementary school in New York	15,768	1) SMS reminder + school located vaccination	Usual care (autodial reminder and consent packet) and school located vaccination	Influenza vaccination coverage within 6 months	No difference between SMS reminder group and usual care groups (4.4% vs 4.3%)	High opt-out rate. No mention to randomisation concealment or blinding procedures. No mention of how sample size was calculated	Good
Stockwell et al. (2015) [42]	USA: Low-income, urban, minority (Latino) population presenting for 1st flu vaccine between 6–18 months of age in New York	660	1) SMS scheduling reminder + written reminder; 2) SMS educational + written reminder	Written reminder only	Vaccination coverage: receipt, and Vaccination timeliness of second dose of influenza vaccine	Educational SMS arm more likely to receive 2nd dose (72.7% vs 66.7% vs 57.1%, *p* = -0.03) and have timely receipt (*p* < 0.001)	Low-income minority population may limit generalisability	Good
Stockwell et al. (2012) [43]	USA: Low-income, mostly Latino parents in New York	9213	Up to 5 weekly SMS educational & scheduling reminders	Routine care	Vaccination coverage: receipt of influenza vaccine	Higher proportion in intervention group (43.6%, *n* = 1653 vs. 39.9%, *n* = 1509; RRR 1.09 95% CI 1.04–1.15, *p* = 0.001)	Possible selection bias as there were 8% of siblings allocated to opposite group	Good
Stockwell et al. (Text4Health: Adol) (2012) [44]	USA: Low-income parents of under-vaccinated adolescents (11y-18y) in New York	361	SMS reminder	No reminder	Vaccination coverage: meningococcal and Tdap dose uptake	More adolescents in intervention group received meningococcal & Tdap at 24 weeks compared to control (36.4% vs. 18.1%, *p* < 0.001)	Potential under-reporting of vaccination receipt. Randomly selected intervention and control groups from cohort instead of traditional 1:1 randomisation procedure. Unsure if blinding occurred to negate performance bias	Poor
Stockwell et al. (Text4Health: Paeds) [44]	USA: Low-income parents of under-vaccinated children (7mo-22mo) in New York	174	SMS recall + letter	Letter recall	Vaccination coverage: Hib vaccine uptake	More children in intervention group received Hib vaccine compared to control (21.8% vs. 9.2%, *p* < 0.05)	Unsure if blinding occurred to negate performance bias. Low sample size	Fair
Tull et al. (2019) [4]	Australia: Parents of adolescents due for HPV vaccine	4,386	1) Motivational (educational) SMS; 2) Self-regulatory SMS	No SMS reminder	Vaccination coverage: HPV vaccine uptake	Both SMS reminder group similarly effective to improve vaccination rates (88.35% vs 89) compared to control (85.7%, *p* < 0.016)	Sample skewed towards metropolitan schools. Adolescents had to consent to receive a vaccine before being sent a reminder	Good
Wiseman et al. (2016) [45]	USA: Parents of children attending a primary care clinic in Arizona	136	SMS reminder	Sham health-related SMS	Vaccination coverage: influenza vaccine uptake by end of influenza season	More children in SMS reminder group received vaccine compared to sham SMS (83.5% vs 45.4%). OR: 4.46, 1.704–11.706, < 0.001)	Small convenience sample and potential selection bias. Randomisation procedures not adequately described	Poor

*Abbreviations:* *AHR* Adjusted hazards ratio, *AOR* Adjusted odds ration, *BCG* Bacillus Calmette–Guérin, *CI* Confidence interval, *DPT* Diphtheria pertussis tetanus, *Hib* Haemophilus influenzae type b, *HPV* Human papilloma virus, *HR* Hazard ratio, *mCCT* Mobile conditional cash transfer, *mo* Month, *OR* Odds ratio, *RR* Risk ratio, *RRR* Relative risk reduction, *SMS* Short message service, *wk* Week

### Study setting and participants

Of the selected trials, 19 were conducted in high-income countries and 11 were conducted in low-middle income countries. Sixteen of 19 trials conducted in high-income countries were limited to the United States and targeted parents of children from low-income or ethnic minority groups, and children attending tertiary-affiliated, private paediatric clinics or local hospitals *(*Table 1*).* One study targeted parents attending a local baby exhibition event [36]. Trials in low-middle income countries were conducted in Nigeria (3), Kenya (2), India (2) Guatemala (2), Pakistan (1), Zimbabwe (1). In the trials from high-income countries, the SMS-based intervention recipients were predominantly English-speaking and female. Maternal education levels were more commonly reported among trials conducted in low-middle income countries.

### Interventions and comparator

The SMS-based interventions were compared against a range of comparators ranging from routine care (no SMS or reminder) (16), written reminders (7), telephone reminders (either from practice staff or automated calls) (3), sham or health-related SMS-based reminders unrelated to vaccination (3) health education (1). Two RCTs compared the effectiveness of SMS-based reminders when combined with monetary or phone credit incentives compared to SMS-based reminders alone, or other strategies [6, 28].

### Study quality

Please see Table 1 for individual study quality and risk of bias assessment. Seventy percent (21/30) of the trials were deemed to be of fair to good quality. The most frequently identified sources of bias were related to poor or poorly documented randomisation procedures, or a lack of adequate detail regarding allocation concealment or blinding of practice staff.

### Effect of SMS-based interventions on vaccine coverage

#### Low-middle income countries (LMIC)

Eight of ten trials conducted in LMICs reported higher vaccine coverage among children of parents who received SMS-based reminders compared to non-SMS interventions or routine care *(see *Table 1) [20, 21, 23–27, 29]. Two of ten trials found no evidence of an effect of SMS-based reminders alone on vaccine coverage compared to no SMS, but found evidence of a small effect when SMS-based reminders were combined with a monetary incentive [6] or phone credit incentive [28]. One trial found evidence that SMS-based reminders were more effective than control (no SMS reminder), and that the effectiveness of SMS-based reminders was greater when combined with incentives in the form of high phone credits [21].

#### High income countries (HIC)

Ten of 17 trials conducted in HICs [34, 35, 37, 38, 42–46] reported small or modest improvements in vaccine coverage among children of parents receiving SMS-based reminders compared to those who received no SMS or alternative non-SMS strategies; the remaining 7 trials [30, 32, 33, 36, 39, 41, 47] found no evidence of an effect of SMS-based intervention compared to non-SMS control (appointment cards, alternative health messages or no reminder).

Two of the 17 trials reported improvements that were limited to specific timepoints or in specific recipient groups, but not all [33, 35]. One of the 2 trials found evidence that SMS-based reminders were effective compared to control (no SMS reminder) for vaccines scheduled at 12 months-old only, with the effect slightly greater when SMS-based reminders were combined with a personalised calendar; a post hoc analysis found evidence of a greater effect among children who had been late for any previous vaccine [35]. The second trial reported no overall difference between groups (SMS reminders and SMS vaccination appointment reminder, SMS vaccination appointment reminder only & control) for receipt of MMR vaccination, but a sub-group analysis reported a difference for parents who did not have an appointment prebooked in the SMS reminder and appointment reminder arm compared to SMS only and control [33].

Regarding message content, six of the 17 trials compared SMS-based reminders with embedded educational/persuasive content; of these 5 found evidence of increased vaccine coverage compared to plain SMS-based reminders without these features [34, 37, 42, 43, 46].

Of the 17 studies, one trial reported improved vaccine coverage among parents receiving reminders through interactive messaging (ability to exchange bidirectional messages or receive further information) compared to no SMS reminder [38], and one reported interactive messaging in combination with educational SMS reminders resulted in higher coverage compared to educational SMS only or telephone reminders [34].

#### SMS-based interventions: pre-call and recall vaccine reminders

In 16 trials, SMS-based reminders were issued prior to vaccine due-dates; of these 12 found evidence that vaccine coverage was higher in the SMS-based intervention group than the comparator group [6, 20, 21, 23, 25–27, 29, 34, 43, 45, 46]. Among the 4 trials that found no evidence of a difference in coverage [30, 33, 41, 47], two trials reported significant implementation problems in the intervention group including a high rate of failed SMS delivery [30, 33].

In 6 trials [24, 34, 38, 43, 44], SMS-based reminders were issued to parents whose children were already overdue for receipt of a recommended vaccine; all found evidence that vaccine coverage was higher in the SMS-based intervention group compared to control.

In 3 trials both pre-call and recall SMS-based reminders were used [27, 35, 37]; two trials reported improved vaccine coverage in the intervention groups compared to control [27, 35], and one reported that receipt of an SMS-based reminder was only effective if it contained an educational message [37]. No trials directly compared pre-call to recall message strategies.

#### Special interest groups/vaccinations

Four trials [32, 38, 39, 44] examined the effect of SMS-based reminders for adolescent vaccines, including HPV and meningococcal vaccines; two trials [38, 44] reported evidence of higher vaccine coverage among SMS-based reminder recipients (parents in 4 studies, and either parent or adolescent in 1 study) compared to no SMS, other comparator groups, or historical control.

### SMS-based interventions and timeliness of vaccination

#### Low-income settings

All five trials [6, 20, 22, 26, 28] conducted in low-income countries found evidence of improved vaccine timeliness in children of parents receiving SMS-based reminders compared to control. One trial reported that compared to a control group, there was improved vaccine timeliness in groups who received an SMS-based reminder either with or without a monetary incentive [6]; the other trial did not find evidence that SMS-based reminders alone improved timeliness, but found evidence that an SMS-based reminder plus a phone credit incentive did improve timeliness compared to control [28]. One trial found evidence that standard SMS and educational SMS-based reminders had a similar and superior effect on vaccine timeliness compared to control (no reminder), but phone call reminders appeared to be more effective than either SMS-based intervention [26].

#### High-income settings

Of 7 trials that reported on vaccine timeliness, 5 found evidence that SMS-based reminders improved vaccine timeliness compared to standard care [31, 33, 34, 40, 42]. One trial reported that compared to a standard SMS-based reminder or non-SMS control, a higher proportion of children whose parents received an educational SMS-reminder received a timely second dose of influenza vaccine; there was no difference in timeliness between the standard SMS and control [42].

## Discussion

Compared to alternative strategies to try to improve vaccine coverage and timeliness, SMS-based strategies are instantaneous, convenient, scalable, have potential for automation, and are relatively low cost [48]. We found evidence that they can be effective in both low-middle- and high-income country settings, but where effect was observed, it was usually small to moderate in size, with the greatest observed effect for vaccine coverage being a risk ratio of vaccination of 1.36 *(see *Table 1*).*

The SMS-based interventions evaluated varied in several respects; some included educational content, some were combined with incentives, and some were delivered as recall rather than as pre-call reminders. The apparent effectiveness of these strategies varied across settings; for example, one of the more robust LMICs studies reported that SMS combined with airtime incentives were most effective for parents in Pakistan compared to SMS only and no reminder [21]. The three largest trials examining the effectiveness of SMS-based reminders on vaccine uptake, found no evidence of an effect compared to control [32, 41, 47]. Baseline vaccine coverage was low in these trials, and we note that none of these studies used SMS reminders with educational or persuasive content. We speculate that plain SMS-based reminders might only be effective where population acceptance of vaccination is already high. No trials were identified which directly compared pre-call to recall SMS-based reminders.

The differential effect of SMS-based interventions across socioeconomic groups within the same setting has not been extensively studied. In many settings, children from low income families have lower rates of vaccine coverage [49]; reduced health literacy and logistical barriers such as poor access to primary healthcare have been reported as potential contributing factors [50, 51]. SMS-based reminders may be effective for families with limited access to other forms of communication, such as email [52]; however, some studies have reported specific barriers to SMS in families with low-socioeconomic status, including unreliable service delivery [23] and changing contact details and service providers [30]. In some settings mobile phone service providers require the SMS recipient to have sufficient credit to receive messages; this may not be relevant to all settings.

We sought to understand whether there would be observed differences in the impact of SMS reminders across different contexts, including childhood and adolescent vaccinations. There was a paucity of evidence assessing impact of reminders on adolescent vaccinations; two of the four studies reported improvements, however only one study was considered good quality [38]. Among the studies that reported higher vaccination uptake, these improvements were broadly comparable to improvements observed in trials in childhood vaccination (up to 30% in coverage). No studies directly compared the effectiveness of SMS reminders delivered to adolescent recipients versus their parents, which would be helpful to ascertain which is most effective, and whether different messaging strategies for each are required.

It can be difficult to know whether an SMS has been received, read, and understood by the intended recipient. Bidirectional messaging, wherein SMS messages are sent back-and-forth between the recipient and the vaccine provider, may be used to confirm receipt of the message and/ or understanding of its content, or to provide supplementary educational material to parents prior to vaccine appointments. While we identified some evidence of the effectiveness of bidirectional messaging in two trials [34, 38], the cost and burden on providers to issue more personalised messaging needs to be considered.

We identified evidence that SMS-based reminders had improved efficacy where the messages included educational content, especially for vaccines that may not be part of a routine vaccine schedule, such as for influenza vaccine. Trust between parents and vaccine providers has been identified as important in preventing vaccine hesitancy [53]. This may indicate that educational or persuasive SMS reminders from providers that have a strong and trusting relationship with families may be a determinant of vaccination behaviour.

SMS-based strategies may represent an opportunity to directly address adverse vaccine beliefs through educational messaging. However, more research is needed to determine what educational content and message framing is most effective (e.g. benefit versus risk-based message framing). Many SMS services impose a message character limit, so achieving a message with sufficient content to motivate action is a challenge [34]. There may be benefit to developing educational content which is based on behavioural theories or frameworks such as the health belief model [54].

### Strengths and limitations

This review included trials across a range of contexts, including high- and low-middle income country settings. We also included vaccine timeliness as an outcome of interest as prior research has indicated that important delays in vaccine receipt may exist even in settings with high vaccine coverage [55–57]. Timeliness was less frequently reported as an outcome than vaccine coverage. The decision for a narrative review has limited our ability to summarise the effect size of SMS-based interventions. Meta-analysis was not suitable due to the vast heterogeneity of the interventions, contexts of the studies, and the outcomes measured and reported.. We only included trials in this review, although we note that a number of observational studies have reported on the post-implementation impact of SMS-based reminders, and these might provide additional insights into the apparent heterogeneity in effects.

## Conclusions

We found evidence that SMS-based reminders can have a beneficial effect on the coverage and timeliness of routine vaccines in childhood across a range of LMIC and HIC settings. We found some weak evidence of the effectiveness of educational versus standard (non-educational) SMS message content, and for an additional effect of monetary or phone credit incentives, although more studies are needed to corroborate these findings. No studies directly assessed the effect of pre-call versus recall timing of messages. As such, neither the optimal message content (i.e. plain versus educational/persuasive) nor optimal timing of SMS-based reminders have been clearly determined. Multi-arm or factorial-design trials evaluating alternative options for SMS content and timing in varying combinations and across different age groups and programmatic contexts could help to address these gaps [58]. Trials should also assess their cost-effectiveness when delivered as vaccine pre-call versus recall, or in the context of targeted and possibly multifaceted strategies which are tailored for specific populations.

### Supplementary Information

Supplementary Material 1.

## Data Availability

All data analysed in this review has been provided in the published article.

## Abbreviations

CINHAL: Cumulative Index to Nursing and Allied Health Literature
HIC: High income countries
HPV: Human papilloma virus
LMIC: Low-middle income countries
MMR: Measles mumps rubella
NHLBI: National Heart Lung Blood Institute
PICO: Participants Intervention Comparator Outcome
RCT: Randomised control trial
SMS: Short Message Service
