# Supplementary material for: SMS-based interventions for improving child and adolescent vaccine coverage and timeliness: a systematic review
Source: BMC Public Health. 2024 Jul 2;24:1753. doi: 10.1186/s12889-024-18900-4 (PMC11218178; doi:10.1186/s12889-024-18900-4)
Supplement: Supplementary file 1 — Supplementary Material 1. [file 12889_2024_18900_MOESM1_ESM.docx]

***Appendix 1: Electronic search strategy***


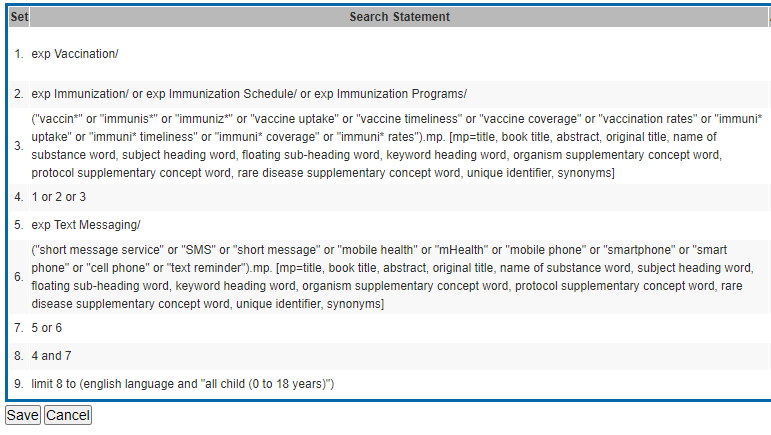


Abbreviations: exp, explore; mp, map heading; SMS, short message service.
